# An Optimized Tightly-Coupled VIO Design on the Basis of the Fused Point and Line Features for Patrol Robot Navigation

**DOI:** 10.3390/s19092004

**Published:** 2019-04-29

**Authors:** Linlin Xia, Qingyu Meng, Deru Chi, Bo Meng, Hanrui Yang

**Affiliations:** 1 School of Automation Engineering, Northeast Electric Power University, Jilin 132012, China; 13846877678@163.com (Q.M.); ANATKH237@163.com (D.C.); yanghanrui1208@163.com (H.Y.); 2 School of Computer Science, Northeast Electric Power University, Jilin 132012, China; mengbo_nannan@163.com

**Keywords:** tightly-coupled VIO, SLAM, fused point and line feature matching, pose estimates, simplified initialization strategy, patrol robot, map representation

## Abstract

The development and maturation of simultaneous localization and mapping (SLAM) in robotics opens the door to the application of a visual inertial odometry (VIO) to the robot navigation system. For a patrol robot with no available Global Positioning System (GPS) support, the embedded VIO components, which are generally composed of an Inertial Measurement Unit (IMU) and a camera, fuse the inertial recursion with SLAM calculation tasks, and enable the robot to estimate its location within a map. The highlights of the optimized VIO design lie in the simplified VIO initialization strategy as well as the fused point and line feature-matching based method for efficient pose estimates in the front-end. With a tightly-coupled VIO anatomy, the system state is explicitly expressed in a vector and further estimated by the state estimator. The consequent problems associated with the data association, state optimization, sliding window and timestamp alignment in the back-end are discussed in detail. The dataset tests and real substation scene tests are conducted, and the experimental results indicate that the proposed VIO can realize the accurate pose estimation with a favorable initializing efficiency and eminent map representations as expected in concerned environments. The proposed VIO design can therefore be recognized as a preferred tool reference for a class of visual and inertial SLAM application domains preceded by no external location reference support hypothesis.

## 1. Introduction

When robots operate under an unknown environment, an absolute external location reference such as a Global Positioning System (GPS) may be not available, and the no-prior-knowledge based navigating technology will be highly required. Thus, the individual intelligent robot should have the ability to estimate its own location using the carried sensors, such as Inertial Measurement Units (IMUs), laser radars, cameras, et al. [1,2,3]. For the navigation and perception problems of patrol robots working in the substations, the electromagnetic interferences will influence the signal transmissions, which therefore does not allow for the GPS receiver to assist the patrol robots with continuous and steady signal supports. In contrast to the existing navigation modes performed by dedicated external sensors, the robust solutions mainly lie mainly in utilizing the essential visual functions of cameras to build an environment map in real-time and estimate the position of the robot within the map simultaneously. This problem is called simultaneous localization and mapping (SLAM). It is noteworthy that SLAM may not only contribute to the acquisition and identification of the scene knowledge by some appropriate mode, but that it may also improve navigation performances with steady pose estimates [4]. One of the most significant SLAM results is proposed by Davison A.J., who pioneered the updating of the states of cameras and landmark points by an extended Kalman filter (EKF) and addressed the real-time SLAM problems for practical applications [5]. Klein G. extended the above model using a nonlinear optimization. He explicitly structured the SLAM system in terms of the front-end and the back-end, and improved the matched back-end framework by having the fused global constraints of the state variables be optimal rather than the pure iterations of EKF [6].

The above methods form the basis of feature-based methods for an efficient pose estimation [7,8,9]. Under the simple circumstances where the illumination changes slowly, or the cameras equipped are at a low speed movement, the direct methods are generally simpler to apply in practice, directly recovering the camera motion by minimizing a pixel-level intensities-based measurement error with no need to detect feature points [10,11,12]. Lately, there has been more research in the area of SLAM-based robot localization. In cases where the accurate pose estimates and large-scale scene reconstructions for mapping tasks are desired, the feature-based methods are more suitable for robotic applications.

Some research focuses on eliminating the accumulative positioning errors mainly caused by the incorrect feature points matching among images [13,14]. Actually, considering the fact that the cameras in motion find it difficult to present the expected brilliant images continuously, and in view of the fact that in some cases the cameras are working under the scenes with poor visibility or the ‘understanding’ of scenes can not be achieved in terms of textures, a visual inertial odometry (VIO) scheme is generally preferred, by fusing the inertial recursion (IMUs present) and SLAM calculation (cameras present) in robotics, to satisfy a long-term positioning accuracy and a matched favorable navigation stability in a short-time rapid maneuver.

By a method in which the state of the camera and the state of IMU are either directly incorporated in one state estimator or not, the typical VIO may be classified into a loosely-coupled mode and tightly-coupled mode. A loosely-coupled VIO separately estimates the relative motion by two state estimators, viz., the state of the camera and the state of IMU are separately estimated, and the VIO makes a fusion of these two results. A tightly-coupled VIO fuses raw measurements from the camera and IMU, explicitly estimating the relative motion by one state estimator, and this is generally fulfilled by constructing the joint nonlinear loss functions associated with the state variables. By contrast, the tightly-coupled mode presents a better accuracy and robustness.

For the state estimation, a filter-based method and optimization-based method are both possible [15,16,17,18]. The tightly-coupled mode fully takes into account the coupling between the used sensors. The optimization-based method explicitly incorporates the raw measurements of sensors and globally optimizes the sensor states by one estimator. As a mainstream framework, the tightly-coupled optimization-based VIO has been greatly extended theoretically. In principle, the system state of a VIO is expressed by typically integrating the pose (such as a rotation and translation by IMUs/cameras), velocity and zero bias (such as an inherent gyro bias and accelerometer bias by IMUs). The system state estimation of a VIO can converge to the desired state by optimizing the previously-constructed loss functions with respect to the state. It should also be noted that the initial values of the state variables for the global optimization are given by a system initialization module. To guarantee the long-term and steady availability in cases where limited numbers of feature points or textures are present, some research has been developed to improve the feature extraction pattern by fusing the line features or plane features in the VIO front-end, enabling the cameras to efficiently keep tracking. These solutions are equivalent to exerting some additional constraints to the entire pose estimation tasks [19,20].

The maturation and development of the above techniques underpin a successful robot application in the power patrol inspection. Accordingly, the efficiently initialized VIO permits the robot to perform accurate localization and navigation tasks [21,22]. Based on the above discussion, an optimized VIO system is presented to take into account the problems associated with the initialization efficiency and feature matching results.

The main contributions to this paper are shown in the following aspects.

1. First, during the course of a VIO initialization, the constant-velocity constraints are applied to the robots in motion. The consuming time for calculating the camera rotation between frames, is, in consequence, much less than that under the non-restriction conditions, accelerating the acquisition process of the initial state variables (including the pose, velocity, zero bias, etc.) dynamically.
2. Second, as a consequence of explicitly taking into account the textures of the electrical equipment in the work volume, the improved VIO characterized by the feature matching in terms of point features and line features enables the camera movement estimation (such as the rotation or translation) to be more accurate and smooth.
3. Third, the sparse maps represented by the point features and line features are constructed as expected under the sliding window optimization model. The introduction of this practical optimization model improves the efficiencies of the state estimation and mapping. Additionally, both dataset tests and substation scene tests for the robot routing inspection applications have been conducted, and the detailed evaluation results are given.

The outline of the remainder of the paper is as follows. The following section mainly discusses the VIO anatomy, besides the detailed description of the VIO front-end, including the reprojection errors associated with the points features and line features; additionally, the IMU pre-integration model is given, and the superiority of the fused-point and line feature-matching based method in accurate pose estimates over the direct method and simple point feature-matching based method is numerically proven by multiple sets of simulations. In Section 3, a simplified VIO initialization strategy is proposed and discussed, which subsequently includes a gyro bias estimation, accelerometer bias and gravity estimation, and scale factor and velocity estimation; furthermore, the laboratory test on the comparative time consumption by three typical feature-based visual odometries (VO) is highlighted. The matched state variable optimization tasks in the VIO back-end are emphasized in Section 4; specifically, the sliding window model for the accumulated error reduction and the visual measurement model for the two Jacobian matrix calculations with respect to the reprojection errors defined in Section 2, are respectively established. Section 5 carries out the experiments on dataset tests and real substation scene tests, and presents the main conclusions of this investigation.

## 2. Overall Description of Tightly-Coupled VIO

The physical structure of VIO can be divided into two parts: an IMU and a monocular camera. The embedded IMU provides the VIO system with an orthogonal 3-axial acceleration and angular rate in the body (robot) coordinate frame. The camera is mounted on the stationary base of the robot, providing the VIO system with sequential image information, by which it estimates the robot pose in the world coordinate frame and which can be further applied to represent and address the structure from motion (SFM) problem [23,24]. The essential part of integrating these two components consists in updating the state variables of the tightly-coupled VIO system as time evolves, so as to efficiently obtain the global optimum solutions of the state variables.

### 2.1. VIO Anatomy

**Figure 1:**
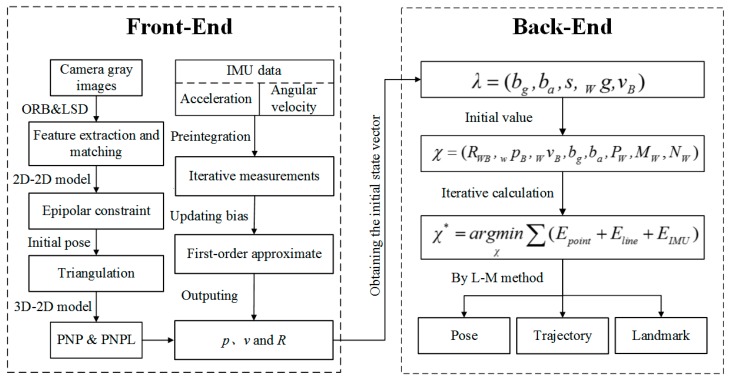
Flow chart of a VIO state estimator algorithm.

Denote the world coordinate frame of the VIO system by W, which is referred to as the absolute reference used to denote the position and orientation of the objects in the concerned scenes. Denote the IMU coordinate frame (body coordinate frame) and the camera coordinate frame by B and C, respectively. A transformation between W and B is represented by a homogeneous transform matrix TWB=(RWB|pWB), where $R_{WB}^{}$ represents the rotation and pWB represents the displacement. Let vWB denote the robot velocity expressed in the world coordinate frame. Denote the gyro bias and accelerometer bias by $b_{g}$ and $b_{a}$, respectively. Figure 1 presents the diagrammatic representation of a VIO state estimator algorithm.

As illustrated, Figure 1 shows how information flows forward from the front-end to the back-end of the process. The VIO front-end collects the manipulated inputs from the IMU and the camera, and after obtaining the raw pose estimates of the robot in motion it turns to the VIO back-end to calculate the initial state vector λ. As mentioned above, the fused point and line feature-matching based method is conducted for the ideal pose estimates, on basis of the gray images.

The VIO back-end is used to optimize the state vector χ from λ. Let:

(1) λ=(bg,ba,s,gW,vWB)χ=(RWB,pwB,vWB,bg,ba,PW,MW,NW)χ∗=arg minχ∑k(Epoint+Eline+EIMU)

where s represents the scale factor of the monocular camera, and gW represents the gravity vector expressed in the world coordinate frame. χ represents the VIO state vector and $\chi^{*}$ represents the loss function with respect to χ. $P_{W}^{}$ and $\left(M_{W}^{},N_{W}^{}\right)$ respectively represent the point features and line features of the images in the world coordinate frame. $E_{point}$ and $E_{line}$ are, respectively, the constructed quadratic form functions of the point feature reprojection error and line feature reprojection error. $E_{IMU}$ is also a quadratic form function of the IMU error, which in nature denotes the constraints between the current frame and the previous keyframe in terms of a series of variable errors, like the rotation, position, velocity and bias [25]. Minimize the loss function $\chi^{*}$ by means of a typical Levenberg-Marquardt iterative calculation to assure the global optimization results, viz., the VIO can put out the globally optimal pose, trajectory, and landmark position in the world coordinate frame.

Note that the relative position and orientation between the camera and the IMU are fixed once the installation is done. Analogously, the transformation relationship between C and B can be represented by a homogeneous transform matrix TCB=(RCB|pCB), where $R_{CB}^{}$ represents the rotation and pCB represents the displacement. More specifically, $T_{CB}$ essentially has a major impact on the precision and stability of the VIO system, which should therefore be calibrated with some mathematical means beforehand. Referring to the existing well-developed ways [26], the typical hand-eye calibration method is adopted in this paper.

### 2.2. Reprojection Error of the Camera

As described above, the VIO system fuses the point features and line features derived from the camera images. For the point features, the reprojection error denotes the distance (on the imaging plane) of the projection position of 3-D points from the detected position, minimizing this error by means of identifying the matched transform matrix, which then indicates that the pose optimization process is fully implemented. Suppose $P_{i}=(X_{i},Y_{i},Z_{i})$ is the position of the ith feature point in 3-D space and $u_{i}$ is the detected projection position of $P_{i}$ on the imaging plane, the constructed reprojection error in terms of the point features can be defined as [27]:

(2) $$r_{point}=u_{i}-\frac{1}{z_{i}}Kexp(\xi^{\wedge })P_{i}$$

where, $z_{i}$ is the depth of $P_{i}$, and K is the intrinsic matrix of the camera. ξ is the Lie algebraic representation of the pose, and it follows that:

(3) $$\xi^{\wedge }=\left[\begin{array}{ccc} 0 & -\xi_{3} & \xi_{2}\\ \xi_{3} & 0 & -\xi_{1}\\ -\xi_{2} & \xi_{1} & 0 \end{array}\right]$$

**Figure 2:**
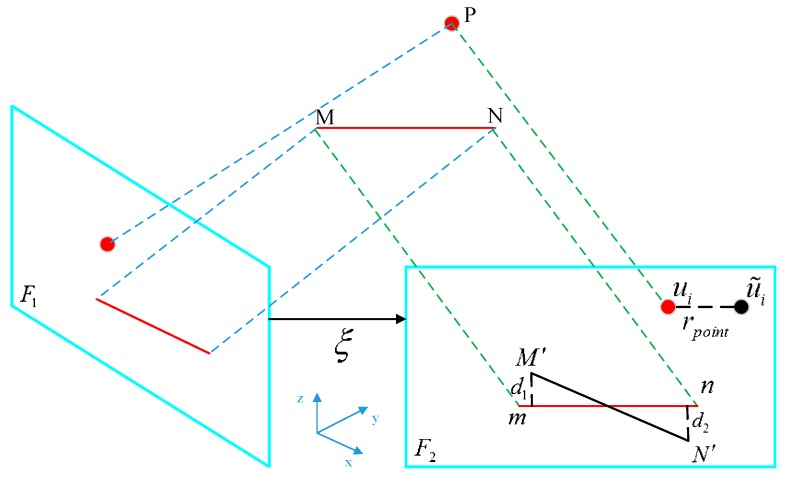
The graphic interpretation of the point/line feature reprojection error.

For a line segment with the ends $M,N\in R^{3}$, the line reprojection error denotes a sum of point-to-line distances between the projected line segment l ends (m,n) and the detected line segment $l^{\prime }$ ends ($M^{\prime },N^{\prime }$) on the imaging plane; it follows that [28]:

(4) $$r_{line}(M^{\prime },N^{\prime },l,\xi ,K)=r_{pl}^{2}(M^{\prime },l,\xi ,K)+r_{pl}^{2}(N^{\prime },l,\xi ,K)$$

where, $r_{pl}^{2}(M^{\prime },l,\xi ,K)$ represents the distance between the detected position of $M^{\prime }$ and line l, similarly, $r_{pl}^{2}(N^{\prime },l,\xi ,K)$ represents the distance between the detected position of $N^{\prime }$ and line l. The normalized form l may be defined as:

(5) $$l=(l_{1},l_{2},l_{3})=\frac{m_{d}^{h}\times n_{d}^{h}}{\left|m_{d}^{h}\times n_{d}^{h}\right|}$$

where $m_{d}^{h}$ and $n_{d}^{h}$ respectively indicate the corresponding homogeneous coordinates of the two ends of l. The graphic interpretation of the point/line feature reprojection error is illustrated by the points and line segments in Figure 2.

### 2.3. IMU Pre-Integration

The output frequency of the IMUs is generally dozens of times that of the cameras, which then indicates during the course of the data fusion that the VIO collects multiple sets of IMU measurement data in a single sampling interval [i,i+1] (between two keyframes).

Let ${}_{B}\tilde{a}\left(t\right)$ and ω˜B(t) respectively denote the measured angular rate and acceleration. We have:

(6) a˜B(t)=RBW(aW(t)−gW)+ba(t)+ηa(t)

(7) ω˜B(t)=ωB(t)+bg(t)+ηg(t)

where aW(t) and ωW(t) are the angular rate and acceleration to be estimated. $\eta^{a}(t)$ and $\eta^{g}(t)$ are white noise. The accelerometer bias $b^{a}\left(t\right)$ and the gyro bias $b^{g}(t)$ are subject to random walk noise.

The (i+1)th updated $R_{WB}^{i+1}$, vWBi+1 and pWBi+1 can be given by [29]:

(8) $$R_{WB}^{i+1}=R_{WB}^{i}Exp(({\tilde{\omega }}_{i}-b_{i}^{g}-\eta_{i}^{g})\Delta t_{i,i+1})$$

(9) vWBi+1=vWBi+gWΔti,i+1+RWBi(a˜i−bia−ηia)Δti,i+1

(10) pWBi+1=pWBi+viΔti,i+1+12gWΔti,i+12+12RWBi(a˜i−bia−ηia)Δti,i+12

where $\Delta t_{i,i+1}$ is the time interval between two keyframes. The relative motion between two keyframes can be defined in terms of the pre-integrated $\Delta R_{i,i+1}$, $\Delta v_{i,i+1}$ and $\Delta p_{i,i+1}$, shown as follows:

(11) $$\Delta R_{i,i+1}\dot{=}R_{i}^{T}R_{i+1}=Exp(({\tilde{\omega }}_{i}-b_{i}^{g}-\eta_{i}^{g})\Delta t_{i,i+1})$$

(12) Δvi,i+1=˙RiT(vi+1−vi−gWΔti,i+1)=ΔRi,i+1(a˜i−bia−ηia)Δti,i+1

(13) Δpi,i+1=˙RiT(pi+1−pi−viΔti,i+1−12gWΔti,i+12)=Δvi,i+1Δti,i+1+12ΔRi,i+1(a˜i−bia−ηia)Δti,i+12

Note that it is supposed that bias $b^{a}$ and bias $b^{g}$ are constant during the time interval from t to $t+\Delta t_{i,i+1}$, as indicated in Equations (11)–(13), and for this to be the case they should be initially calibrated in practice. Define the change of $b^{a}$ (and $b^{g}$) as the disturbance δb and linearize it with first-order approximation; consequently, we obtain the (i+1)th state estimates in terms of the *i*th state estimates and the residual error:

(14) $$R_{WB}^{i+1}=R_{WB}^{i}\Delta R_{i,i+1}Exp(J_{\Delta R}^{g}b_{i}^{g})$$

(15) vWBi+1=vWBi+gWΔti,i+1+RWBi(Δvi,i+1+JΔvgbig+JΔvabia)

(16) pWBi+1=pWBi+vWBiΔti,i+1+12gWΔti,i+1+RWBi(Δpi,i+1+JΔpgbig+JΔpabia)

where $J_{\left(\cdot \right)}^{g}$ and $J_{\left(\cdot \right)}^{a}$ are the Jacobian matrices of the pre-integrated measurements with respect to δb at the sampling point *i*.

The pose estimation and IMU pre-integration form the front-end tasks of the designed VIO. To evaluate the performances of the VIO, we carry out a set of numerical simulations. Two images ($F_{1},F_{2}$) derived from fr1/desk of the TUM RGB-D datasets [30] are arbitrarily designated as the testing samples, the fused point and line feature-matching based method and the simple point feature-matching based method, together with the direct method. are conducted under different optimization strategies, including non-optimization, typical Gauss-Newton (G-N) optimization and Levenberg-Marquardt (L-M) optimization for the first round and convergence achieved respectively. The comparative results are shown in Table 1, in terms of the transform matrix $T_{F_{1}F_{2}}$ and RMSE (root mean squared error) values.

As in Table 1, since the direct method estimates the robot pose directly by minimizing a pixel-level intensities-based measurement error, which in nature belongs to the optimization problem, when non optimization is adopted the direct method itself is not available at all. For the first-round G-N optimization, the direct method and the simple point feature-matching based method both fail to result in valid estimates, which is mainly because the trust region problem is not fully taken into account during the optimization process, and consequently an oversized step is employed by mistake. By contrast, the fused point and line feature-matching based method presents a better robustness under a wider range of optimization strategies without any load in complexity; specifically, with the L-M optimization conditions its pose estimation precision is generally best (a lower RMSE between the estimated $T_{F_{1}F_{2}}$ and the true transform matrix given in fr1/desk TUM). The following section concentrates on fulfilling the VIO initialization design for a better state initializing efficiency.

## 3. VIO Initialization Design

The behavior of the VIO highly depends on the initial values of the system states. A proposed method of initializing the VIO states consists of previously setting a constant velocity for a patrol robot in operation. Moreover, it assumes that the rotation is steadily unchangeable. The simplified solution, therefore, is expected to improve the initializing efficiency of an actual VIO without any decrease in the precision. Quite simply, the accuracy of the estimated gravity is evaluated by reference to its true value (since the magnitude of the true gravity is known), so that the effectiveness of the simplified VIO initializing strategy can be verified. The detailed procedures are shown below.

### 3.1. Gyro Bias Estimation

Assume that the relative rotation defined in the pre-integration module is constant, and that the velocity difference is zero during the given time interval [*i*,*i* + 1], [*i* + 1,*i* + 2], …; we have:

(17) $$\Delta R_{i,i+1}=\Delta R_{i+1,i+2},\Delta v_{i,i+1}=\Delta v_{i+1,i+2}=0$$

Define the residual error $r_{\Delta R_{i,i+1}}$ by integrating the terms from the camera calculation and gyro pre-integration. It follows that [31]:

(18) $$r_{\Delta R_{i,i+1}}=\sum_{i=1}^{N-1}Log({\left(\Delta R_{i,i+1}Exp(J_{\Delta R}^{g}b_{i}^{g})\right)}^{T}R_{BW}^{i+1}R_{WB}^{i})$$

where $R_{WB}^{}=R_{WC}^{}R_{CB}^{}$ ($R_{WC}^{}$ is derived from the monocular camera). *N* is the number of keyframes.

The gyro bias $b_{i}^{g}$ is estimated by minimizing $r_{\Delta R_{i,i+1}}$ with the L-M calculation. Among some typical feature point methods such as ORB (Oriented Brief) feature, SURF (Speeded Up Robust Features) feature and SIFT (Scale Invariant Feature Transform) feature, the process of feature extraction and matching cost more execution time. To quantitatively illustrate the time taken for each step of the VIO pose estimation, Table 2 presents the comparative time consumption results through three typical feature-based visual odometries (VO) with a computer Lenovo Y510 (Inteli5-4200MQ, 2.5GHz CPU, 8GB RAM, Lenovo Grope, Beijing, China,) under an Ubuntu 16.04 environment. The images that are used are coming from the fr1_xyz of TUM dataset.

**Figure 3:**
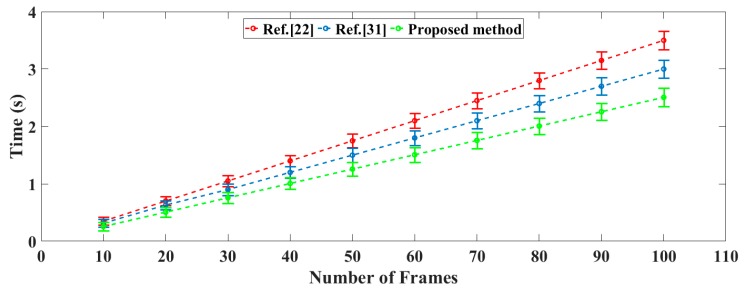
The time consumed for the bias estimation.

As described, the main idea of the VIO initialization lies in calculating the rotation matrix of each frame according to the results from the first two frames on the basis of keeping the rotation constant, rather than repetitively performing a routine feature extraction and feature matching. This is illustrated by the comparative time consumed for the bias estimation in Figure 3; we arbitrarily designate different numbers of the images for testing, and compare the corresponding consumption time by the method in this paper and the typical methods in [22,31]. Clearly, continuously estimating the rotation between the frames reveals its poor efficiency when a larger number of frames are concerned; therefore, the proposed method shows its superiority in dealing with the bias estimation in large-scale scene information.

### 3.2. Accelerometer Bias and Gravity Estimation

The residual error of relative velocity $r_{\Delta v_{i,i+1}}$ may be directly defined on the basis of the constant velocity hypothesis with the known $b_{i}^{g}$, viz., the accelerometer bias is fully taken into account in this case, which is quite different from that adopted in [31]. We define:

(19) rΔvi,i+1=∑i=1N−1(vWBi+1−vWBi︸0−gWΔti,i+1−RWBi(Δvi,i+1+JΔvgbig+JΔvabia))

Analogously, the estimates of the accelerometer bias $b_{i}^{a}$ and the gravity $g_{W}$ are solved by forming a least-square problem with manipulated VIO inputs. It is noted that, in view of the VIO computational load, only three keyframes with a strong parallax excitation are used to establish the fewer simultaneous equations, and this simplified scheme is sufficiently accurate to deal with a wider range of accelerometer bias phenomena.

We further optimize the gravity $g_{W}$ and parameterize it as:

(20) $${\hat{g}}_{W}=g\cdot {\bar{g}}_{W}+\omega_{1}b_{1}+\omega_{2}b_{2}$$

where g is the magnitude of the gravity, and ${\bar{g}}_{W}$ is the direction vector of the current gravity ${\hat{g}}_{W}$. $b_{1}$ and $b_{2}$ are two orthogonal bases on the tangent plane and can be easily determined by the Gram-Schmidt process. $\omega_{1}$ and $\omega_{2}$ are the corresponding 2D components to be estimated. Substitute Equation (20) into Equation (19) and solve it by Singular Value Decomposition (SVD) [32]. This process is iterated several times until ${\hat{g}}_{W}$ converges.

### 3.3. Scale Factor and Velocity Estimation

The scale uncertainty of the monocular cameras may lead to an ambiguous estimate trajectory. The scale factor *s* is therefore introduced to represent the position transformation between the camera and IMU, and it follows that [33]:

(21) pWB=spWC+RWCpCB

Substitute Equation (21) into Equation (16) and ignore the accelerometer bias. We have:

(22) [RWBiT(RWCi−RWCi+1)pCB+12RWBiTgWΔti,i+1+Δpi,i+1]=[RWBiT(pWCi+1−pWCi)−RWBiTΔti,i+1] [svWBi]

Substitute the relative velocity of the pre-integration measurements (expressed in Equation (12)) into Equation (22), and let $\Delta t_{i,i+1}$ and $\Delta t_{i+1,i+2}$ respectively denote the time interval between Keyframe 1 to Keyframe 2 and Keyframe 2 to Keyframe 3. Eliminate the unknown, and we can get ${\hat{z}}_{i,i+1,i+2}$, similar to [31]. Thus, s can be calculated from the residual error equation below:

(23) s∗=argmins(z^i,i+1,i+2−[s(pWCi+1−pWCi)Δti+1,i+2−s(pWCi+2−pWCi+1)Δti,i+1+12gW(Δti,i+12Δti+1,i+2+Δti+1,i+22Δti,i+1)])

In Equation (22), so far, the unknown vWBi is solvable. For the first (K−1) keyframes, the corresponding velocity can be explicitly calculated. Conversely, the current (the Kth) keyframe should be given by Equation (15).

## 4. Tightly-Coupled Information Fusion Based on Sliding Window

The VIO system may proceed, in this phase, by realizing the initialization of the variables illustrated above. The core points consist in continuously optimizing the joint loss functions of each error term (including $E_{point}$, $E_{line}$ and $E_{IMU}$). However, since the front-end of the VIO collects a large amount of input information from the camera and IMU, a heavy emphasis should be placed upon the real-time state estimation of the VIO that has to cope with the potential tracking failures. Considering the computational load in the back-end of the VIO, a practical sliding window scheme is developed to perform the efficient state optimization [34].

### 4.1. Sliding Window Model

The sliding window in the VIO mainly marginalizes out certain states of the system by a Schur complement, and the reinsertion of these as prior information (the prior term $E_{prior}$) would allow the loss functions to be formed and optimized. That is, $E_{prior}$ further supplies the system state with observable constraints. Suppose that the *i*th system state vector (in terms of discrete moment) is χi=(RWBi,pwBi,vWBi,bgi,bai,PWi,MWi,NWi), the matched error terms, can therefore be expressed as:

(24) $$E_{point}=\sum_{k\in K_{V}}\sum_{i\in \beta }\rho (r_{point}^{i,k}{}^{T}\Sigma_{r^{i,k}}^{-1}r_{point}^{i,k})$$

(25) $$E_{line}=\sum_{k\in K_{V}}\sum_{j\in \eta }\rho (r_{line}^{j,k}{}^{T}\Sigma_{r^{j,k}}^{-1}r_{line}^{j,k})$$

(26) $$E_{IMU}=\sum_{i,j\in K_{I}}\left[\rho (r_{\Delta R}^{T}r_{\Delta v}^{T}r_{\Delta p}^{T})\Sigma_{I}{\left(r_{\Delta R}^{T}r_{\Delta v}^{T}r_{\Delta p}^{T}\right)}^{T}+\rho (r_{\Delta b}^{T}\Sigma_{R}r_{\Delta b})\right]$$

where $K_{V}$ and $K_{I}$ respectively represent the sets of visual and inertial measurements in the current sliding window, and $P_{W}^{}$ and $\left(M_{W}^{},N_{W}^{}\right)$ respectively represent the point features and line features which are observed at least twice in the current sliding window. $\Sigma_{r^{i,k}}^{-1}$ and $\Sigma_{r^{j,k}}^{-1}$ respectively represent the information matrix of the point feature reprojection error and line feature reprojection error. $\Sigma_{I}$ and $\Sigma_{R}$ are also information matrices, respectively representing the pre-integration information matrix and bias random walk information matrix. ρ is the robust kernel, piece-wisely expressed as:

(27) $$\rho (s)=\{\begin{array}{cc} \frac{1}{2}s^{2} & \left|s\right|\leq \delta \\ \delta (\left|s\right|-\frac{1}{2}\delta ) & Others \end{array}$$

where ρ(⋅) is in the Huber norm (δ being a pre-set threshold). $r_{\Delta R}$ and $r_{\Delta v}$ are defined in Equations (18) and (19). Analogously, the definitions of $r_{\Delta p}$ and $r_{\Delta b}$ are also derived from the pre-integration measurements, and we have:

(28) rΔp=pWBj−pWBi−vWbiΔtij−12gWΔtij2−RWBi(Δpi,i+1+JΔpgbig+JΔpabia)

(29) $$r_{b}=r_{b}^{j}-r_{b}^{i}$$

The marginalization result can be denoted as the prior term $E_{prior}$, and it follows that:

(30) $$E_{prior}={\left\|r_{prior}-H_{prior}\chi \right\|}^{2}$$

where $r_{prior}$ represents the prior information after marginalization, and $H_{prior}$ represents the Hessian matrix constrained by the pose, landmark position and IMU measurements.

The modified loss function in a linear combination form can therefore be further written as:

(31) $$F_{loss}=\sum_{i}\left(E_{point}+E_{line}+E_{IMU}+E_{prior}\right)$$

**Figure 4:**
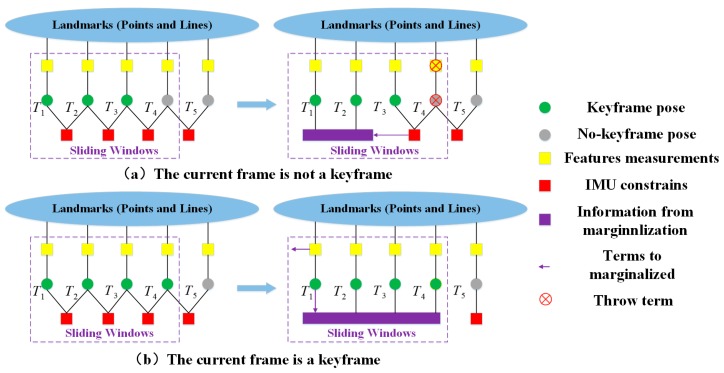
Decision-making pattern of the sliding window model, (**a**) the inserted frame is not a keyframe and (**b**) the inserted frame is a keyframe.

The typical optimization strategy of $F_{loss}$ is similar to Visual-Inertial System (VINS) [35]. Given the frames in the optimization window, the decision-making pattern of the end-back of the VIO is diagrammatically represented in Figure 4. In the figure, the green circle in the figure indicates the pose of the keyframes, the gray circle indicates the pose of the non-keyframes, the yellow square indicates the measurements of the features, the red square indicates the inertial constraints of the IMU, and the purple square and the arrow indicate the information that is marginalized. The red cross indicates the measurements that was discarded. Two cases are discussed: ① if the current inserted frame is not a keyframe, the visual measurement, together with the current pose estimate, would be explicitly neglected, viz., the IMU constraints would only be marginalized out; ② if the current frame is a keyframe, the visual measurement and the pose estimate of the oldest keyframe in the sliding window would be marginalized out and the current keyframe would be kept accordingly.

Owing to the specific forms of the variables to be optimized in the sliding window model, the following work will turn to the definition of the vertices/edges in the graph optimization model by means of a G^2^o optimization framework and to the estimation of the state variables by means of an L-M iterating calculation [36].

### 4.2. Visual Measurement Model

For the loss function represented by Equation (31), the optimization means recurrently performing the linear expansion of Equation (31) around the current estimated value, which therefore implies its principal of calculating the Jacobian matrices of the residual functions with respect to the state variables. Specifically, the method chosen to solve the Jacobian matrix of the point reprojection error with respect to the pose should be the typical chain rule [37], which yields:

(32) $$\frac{\partial r_{point}}{\partial \delta \xi }=-\frac{\partial r_{point}}{\partial P_{C}}\frac{\partial P_{C}}{\partial \delta \xi }$$

with

(33) $$\frac{\partial r_{point}}{\partial P_{C}}=\left[\begin{array}{l} \begin{array}{ccc} \frac{f_{x}}{Z} & 0 & -\frac{f_{x}X}{Z^{2}} \end{array}\\ \begin{array}{ccc} 0 & \frac{f_{y}}{Z} & -\frac{f_{y}Y}{Z^{2}} \end{array} \end{array}\right]$$

(34) $$\frac{\partial P_{C}}{\partial \delta \xi }=[-P_{C}{}^{\wedge },I_{3\times 3}]$$

where δξ is the disturbance of the pose, $P_{C}={\left[X,Y,Z\right]}^{T}$ is the coordinate of the landmark in the camera coordinate frame, and $f_{x}$ and $f_{y}$ are the focal length parameters in K. $I_{3\times 3}$ is an identity matrix.

For the Jacobian matrix of line reprojection error with respect to the pose, let $\ell ={\left[n,v\right]}^{T}$ be the Plücker coordinate of the line feature [38], and let the homogeneous coordinates of $M^{\prime }$ and $N^{\prime }$ be $M^{\prime }={\left(u_{1},v_{1},1\right)}^{T}$ *and* $N^{\prime }={\left(u_{2},v_{2},1\right)}^{T}$ respectively. We have:

(35) $$\frac{\partial r_{line}}{\partial \delta \xi }=-\frac{\partial r_{line}}{\partial l}\frac{\partial l}{\partial \ell }\frac{\partial \ell }{\partial \delta \xi }$$

with

(36) $$\frac{\partial r_{line}}{\partial l}=\left[\begin{array}{ccc} \frac{u_{1}l_{2}^{2}-l_{1}l_{2}v_{1}-l_{1}l_{3}}{{\left(l_{1}^{2}+l_{2}^{2}\right)}^{\frac{3}{2}}} & \frac{v_{1}l_{1}^{2}-l_{1}l_{2}u_{1}-l_{2}l_{3}}{{\left(l_{1}^{2}+l_{2}^{2}\right)}^{\frac{3}{2}}} & \frac{1}{{\left(l_{1}^{2}+l_{2}^{2}\right)}^{\frac{1}{2}}}\\ \frac{u_{2}l_{2}^{2}-l_{1}l_{2}v_{2}-l_{1}l_{3}}{{\left(l_{1}^{2}+l_{2}^{2}\right)}^{\frac{3}{2}}} & \frac{v_{2}l_{1}^{2}-l_{1}l_{2}v_{2}-l_{2}l_{3}}{{\left(l_{1}^{2}+l_{2}^{2}\right)}^{\frac{3}{2}}} & \frac{1}{{\left(l_{1}^{2}+l_{2}^{2}\right)}^{\frac{1}{2}}} \end{array}\right]$$

(37) $$\frac{\partial l}{\partial \ell }=\left[\begin{array}{ccc} \begin{array}{c} f_{y}\\ 0\\ -f_{y}c_{x} \end{array} & \begin{array}{c} 0\\ f_{x}\\ -f_{x}c_{y} \end{array} & \begin{array}{ccc} \begin{array}{c} 0\\ 0\\ f_{x}f_{y} \end{array} & \begin{array}{c} 0\\ 0\\ 0 \end{array} & \begin{array}{cc} \begin{array}{c} 0\\ 0\\ 0 \end{array} & \begin{array}{c} 0\\ 0\\ 0 \end{array} \end{array} \end{array} \end{array}\right]$$

(38) $$\frac{\partial \ell }{\partial \delta \xi }=\left[\begin{array}{cc} -{\left[R_{CW}n_{W}\right]}^{\wedge }-{\left[t_{CW}^{\wedge }R_{CW}v_{W}\right]}^{\wedge } & -{\left[R_{CW}v_{W}\right]}^{\wedge }\\ -{\left[R_{CW}v_{W}\right]}^{\wedge } & 0 \end{array}\right]$$

where v is the direction vector of the line, and n is the normal vector of the plane formed by the line and origin point; they are both in the Plücker coordinate frame. In addition to the Jacobian matrices of the point/line reprojection error with respect to the pose, analogously, the Jacobian matrices of the point/line position in space could be formulized as the similar forms to those in Equations (32) and (35), due to the limits of the space. Please see [39] for details.

## 5. Experimental Section

The experimental observations consist of dataset tests and substation scene tests. The behaviors of the VIO on the datasets largely reflect its actual performances, so the process of evaluating the performances of the designed VIO consists of first testing it in the public datasets.

### 5.1. Dataset Tests and Analyses

The public dataset European Robotics Challenge (EUROC) [40] provides a series of information (such as images, accelerations and angular rates, etc.) invoking a micro aerial vehicle (MAV) equipped with a stereo camera and an IMU in either ① a cluttered workspace scene or ② an industrial machine hall scene. Moreover, the derived information (11 sequences in total) is classified into three grades: “easy”, “medium” and “difficult”, depending for example on the velocity of the aerial vehicle, the texture status of the scene, or the lighting conditions nearby. Also, EUROC presents the standard trajectories captured by the VICON motion capture system with reliable navigating parameters (so-called ‘Ground Truth’) available to users, including the position, attitude, velocity of the MAV in 3D space and some other inertial data, such as the gyro bias and the accelerometer bias obtained by the IMU. Specifically, the V1_01_easy sequence and the MH_04_difficult sequence are designated as the testing samples, and are therefore more appropriate to reflect the strong information domain coverage. In contrast, the state estimates are compared with those extracted by the existing eminent VIOs, such as OKVIS, VIORB, VINS, etc. One thing that should be noted is that, since EUROC doesn’t explicitly provide the Ground Truth scale, we therefore extract it by collecting the translation results from ORB-SLAM2 and translation references provided by Ground Truth. Once we obtained the translation transformation between the first two keyframes in ORB-SLAM2, the truth scale would be a calculation of the translation transformation to the references. Note also that the EUROC dataset presents the stereo images at 20 Hz with IMU measurements at 200 Hz and a trajectory Ground Truth with a higher updating frequency. Hence, the efficient state estimate comparison can only depend upon the accurate alignment of the timestamps. Among these, the VIO trajectory comparison is fulfilled by means of the evo tool [41], and the position error comparison is conducted by the script that TUM provides.

#### 5.1.1. VIO Initialization Results

**Figure 5:**
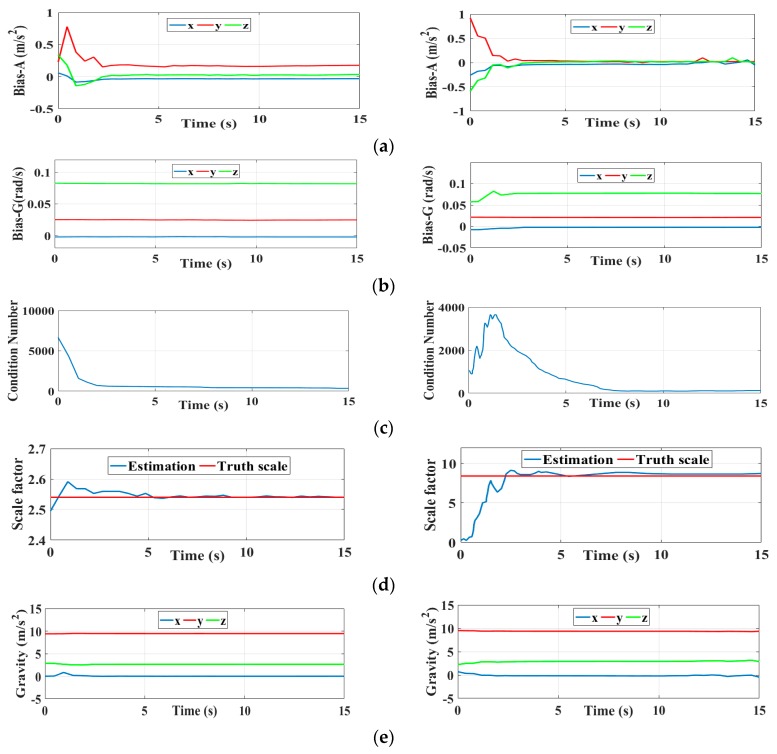
The convergence procedures of the initialization states for the V1_01_easy & MH_04_difficult sequences, (**a**) Initialization results of accelerometer bias; (**b**) Initialization results of gyro bias; (**c**) Calculation of the condition number; (**d**) Initialization results of scale factor; (**e**) Initialization results of gravity vector.

**Figure 6:**
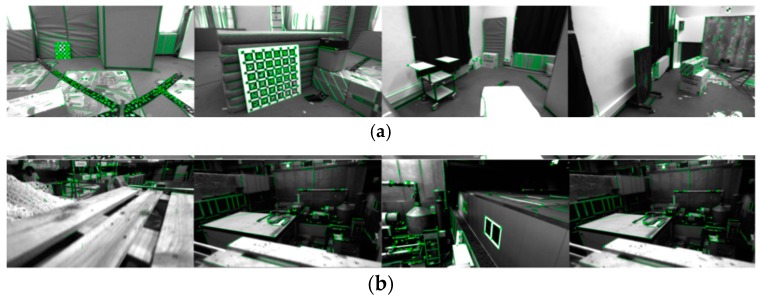
Feature extraction performances of the VIO front-end: (**a**) V1_01_easy sequence; (**b**) MH_04_difficult sequence.

The initialization results are illustrated by the convergence procedures of the initialization state with respect to two typical sequences (V1_01_easy and MH_04_difficult) in Figure 5, and the initialization state is constructed of ① the accelerometer bias, ② the gyro bias in orthogonal tri-axes, ③ the condition number (referring to the data adaptation), ④ the scale factor of the monocular camera, and ⑤ the orthogonal tri-axial component of the gravity vector. Quite clearly, all of these five sets of variables converge for t>8 s. Specifically, the accelerometer bias and gravity vector appear convergent after 2 s, and the accelerometer bias converges to almost zero even under the MH_04_difficult sequence circumstances, while in contrast to this the gyro bias appears larger yet, with more stable characteristics; the reasons for this consist in the fact that we merely calculated and corrected the gyro bias by means of the pose transformation directly derived from the camera, whereas the estimations for the accelerometer bias were implicitly performed by the precise least-square iterations. By comparison, the initialization performances for the MH_04_difficult sequence are slightly inferior, because the condition number illustrated in Figure 6c approximately converges until t=8 s; by then, the observabilities for the initialization state variables are satisfied. Meanwhile, the estimated scale factor, as shown, may be considered to be a true value for t>8 s; the camera trajectory can therefore be recognized as being precisely recovered as expected.

#### 5.1.2. Navigation Performance Evaluations

The feature extraction results are diagrammatically illustrated by Figure 6. As shown, in cases where the scene textures appear clear with an ideal illumination, a large amount of point features and line features are captured as expected (see Figure 6a). Additionally, even though the MH_04_difficult sequence supplies the system with an unstable illumination for representing the MAV in motion circumstances (see Figure 6b), the VIO front-end can still extract enough features and consequently stabilize the dynamic VIO. Here, four representative pictures are selected to describe the scenes that are considered.

The performances of the VIO designed above are diagrammatically given in 3D space, being characterized by absolute positioning errors (APEs). APE is often used as the absolute trajectory error, and the corresponding poses are directly compared between the estimate and reference, and given a pose relation.

**Figure 7:**
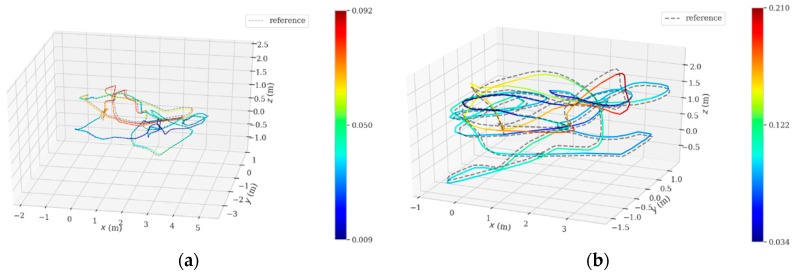

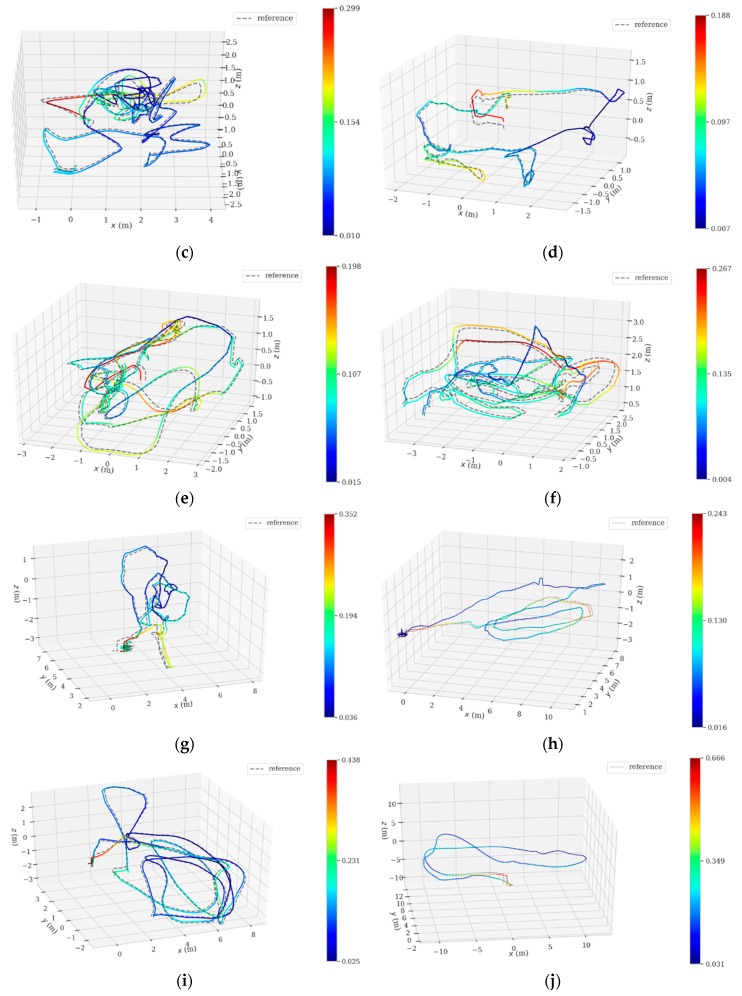

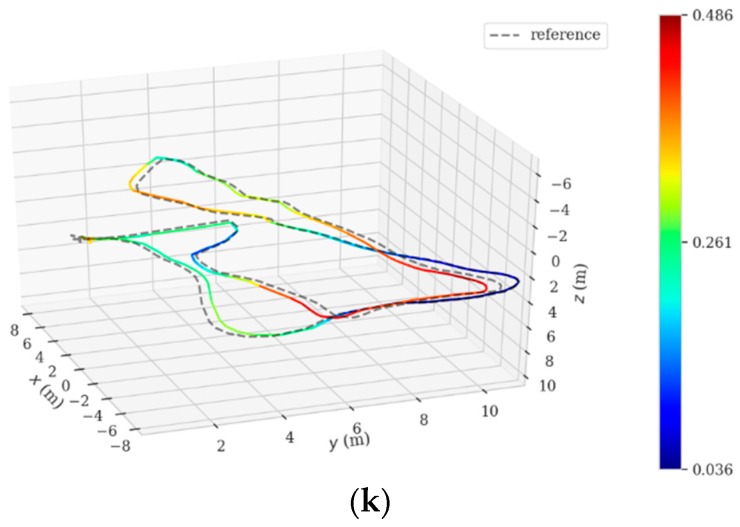
VIO Performances when dealing with sequences at different difficulty levels. (**a**) V1_01_ easy sequence; (**b**) V1_02_ medium sequence; (**c**) V1_03_ difficult sequence; (**d**) V2_01_ easy sequence; (**e**) V2_02_ medium sequence; (**f**) V2_03_ difficult sequence; (**g**) MH_01_easy sequence; (**h**) MH_02_easy sequence; (**i**) MH_03_medium sequence; (**j**) MH_04_difficult sequence; (**k**) MH_05_difficult sequence.

**Figure 8:**
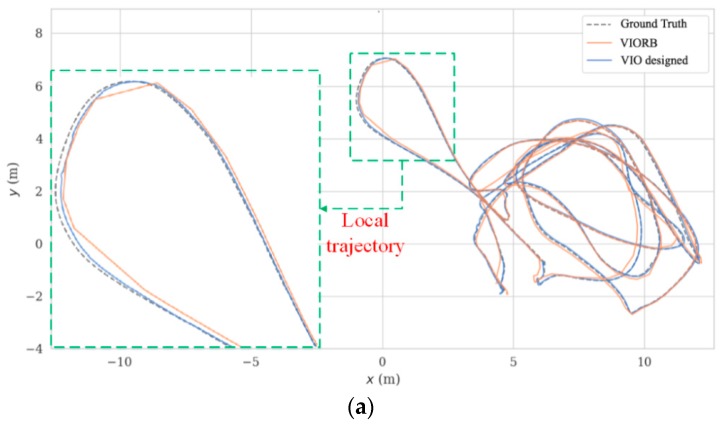

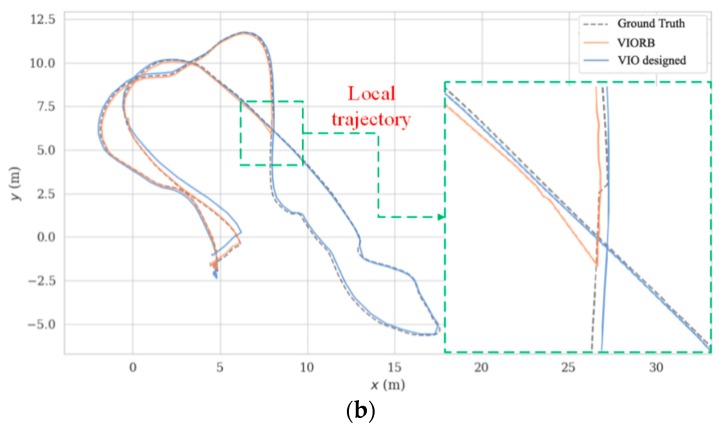
VIO planar trajectory comparisons, (**a**) V1_01_easy sequence; (**b**) MH_04_difficult sequence.

Figure 7a–k corresponds to 11 sequences at different difficulty levels. Furthermore, more detailed analyses related to the two typical sequences (V1_01_easy and MH_04_difficult) are illustrated by planar trajectories, as shown in Figure 8. In Figure 7, the dotted lines represent the Ground Truth trajectories (reference), the color lines represent the estimated trajectories by the designed VIO; the closer the color of the lines approaches to red, the greater the APE, and vice versa. As we can see, the designed VIO presents stable tracking performances for all difficulty levels, even for a fast camera movement or un-ideal illumination circumstances (as V2_03_ difficult and MH_05_difficult denote); no ‘tracking lost’ appears.

The corresponding trajectory comparisons by VIORB (merely with point-based SLAM) and the designed VIO (with fused point and line based SLAM) are given in Figure 8 with a more detailed APE (see Table 3). Considering the fact that the dynamics of the MAV in space are irregular, the 3D trajectory comparisons, would therefore be insufficiently visible; we are, accordingly, mainly concerned with the projected planar trajectory for further analyses (take typical sequence V1_01_easy and sequence MH_04_difficult, for example). In Figure 8, the dotted lines represent the projected Ground Truth trajectories, and the orange full lines and blue full lines respectively denote the trajectories by VIORB and the designed VIO. Figure 8b shows that the VIORB scheme failed to dynamically track the desired Ground Truth trajectory stably. Quite clearly, the orange full line shows its interruption in tracking, which is mainly caused by a lack of environmental textures. Even though the loop closure detection part could help VIORB by restarting the positioning tracing thread according to the previous scene information, the short-term tracking failures could be never acceptable for the actual robot inspection applications. Compared with VIORB, the generated trajectories by the designed VIO kept close to the Ground Truth trajectories (being collected by Vicon). The amplified local trajectories clearly show its superior performances in precision.

**Figure 9:**
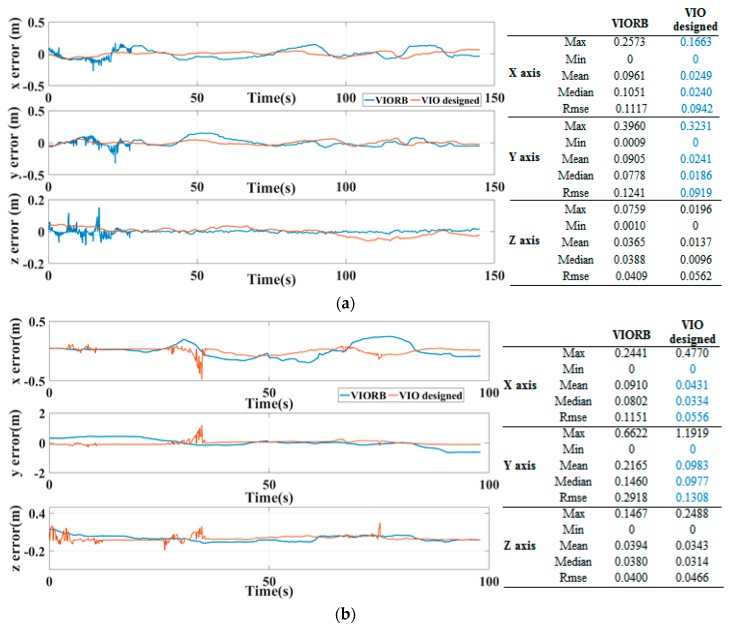
Tri-axial absolute positioning error, (**a**) V1_01_easy sequence; (**b**) MH_04_difficult sequence.

This high precision can also be indicated by the tri-axial APE in the world coordinate frame in Figure 9, and the VIO designed in this paper supplies the combined system with less APE along the X & Y directions in statistics. Two essential enhancements actually facilitate this good result: one is the fused line feature constraints, which further improved the pose transformation precision between the images; the other is the introduced sliding window, which efficiently reduced the data dimension for the back-end optimization. These enhancements are encouragingly achieved with no sacrifices in the VIO operating efficiency.

**Figure 10:**
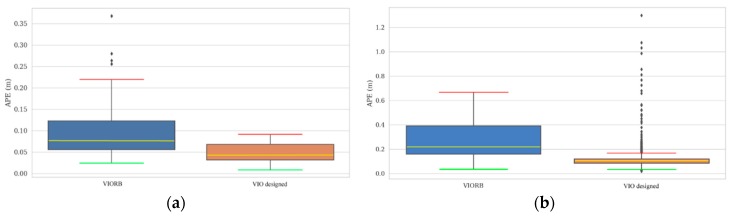
Absolute positioning error distribution, (**a**) V1_01_easy sequence; (**b**) MH_04_difficult sequence.

The corresponding visualized APE distributions are shown in Figure 10a,b, which also statistically shows the max values (red lines), the median values (yellow lines), the min values (green lines) and the concentrated error distributions, being termed ‘mean value domain’ (blue and orange blocks). Here, the remaining points represent the outliers with less weight. As we see, the positioning accuracy by the designed VIO over that by VIORB approaches 4 cm for the V1_01_easy sequence, whose value would be impressively over 16 cm for the MH_04_difficult sequence. Table 3 also gives the detailed APE for the total 11 sequences in terms of the comparison between 5 typical VIOs and the VIO designed in this paper. It can be concluded that the proposed VIO steadily presents its superiorities when dealing with the datasets with different difficulty levels.

#### 5.1.3. Mapping Results

**Figure 11:**
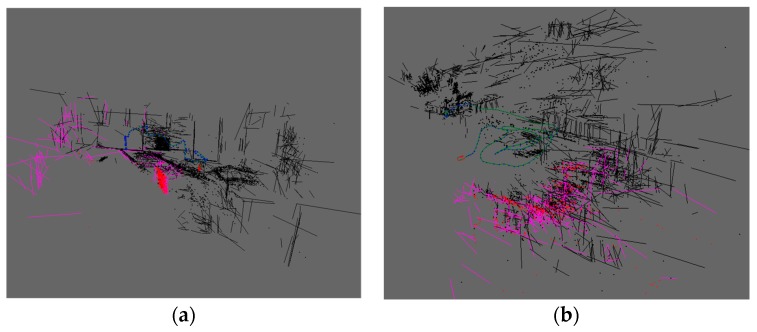
Sparse maps in terms of fused point and line features, (**a**) V1_01_easy sequence; (**b**) MH_04_difficult sequence.

As an illustration of how the point and line features can be fused to support the operations of the VIO front-end, the sparse maps in terms of the fused point and line features for the V1_01_easy sequence and MH_04_difficult sequence are respectively shown in Figure 11. The green lines represent the trajectories of the keyframes, the blue lines represent the selected keyframes for the sliding window optimization, the black points or lines represent the fixed features in 3D space which have been marginalized out, and the red or pink points and lines represent the features which are still in their early optimizing phase. The results indicate that the designed VIO powerfully provides additional structured supports for the typical sparse maps, and this efficient mapping therefore means that it can be recognized as an eminent tool for the solution of scene reconstructions under complex human interaction situations, being preferred for assisting the practical location, navigation and obstacle avoidance tasks.

### 5.2. Substation Scene Tests and Evaluations

The positioning performances are experimentally assessed to evaluate the universal applicability of the VIO designed in practice. The substation scene tests are conducted based upon campus substation (100 m × 40 m rectangle) observations and subsequent laboratory analyses. Table 4 presents the calibration parameters of the camera and IMU we use.

**Figure 12:**
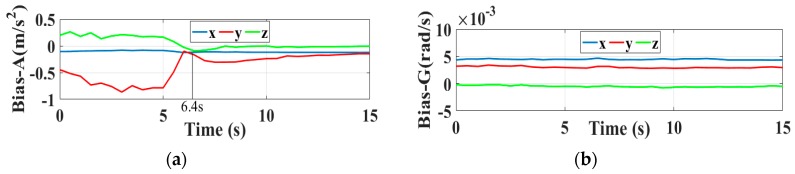

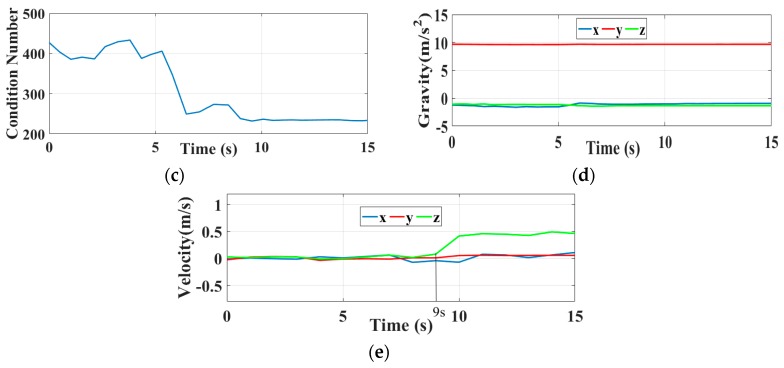
The convergence procedures of the initialization states in the substation scene tests, (**a**) Initialization results of accelerometer bias; (**b**) Initialization results of gyro bias; (**c**) Calculation of the condition number; (**d**) The initialization results of the gravity vector; (**e**) The initialization results of velocity.

Let the robot move around the rectangle with a lower constant velocity; the monocular camera embedded simultaneously entered the working state and was set to initialize the state variables λ by the initialization strategy described in Section 3, once the user workstation obtained the moderate convergent behaviors of the initial state variables. This, then, permitted the robot to perform higher-speed moving tasks (keep walking around the substation). Given the collected information by the user workstation, as shown in Figure 12, the state variables converge for t>6.4 s, as we expected. With a controllable constant velocity, it is relatively efficient to initialize a VIO system. Figure 12e also presents an increase in speed for t>9 s.

**Figure 13:**
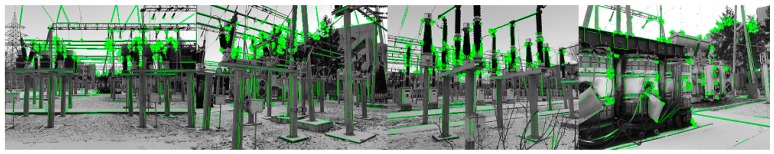
Feature extractions of the VIO front-end in the substation scene.

**Figure 14:**
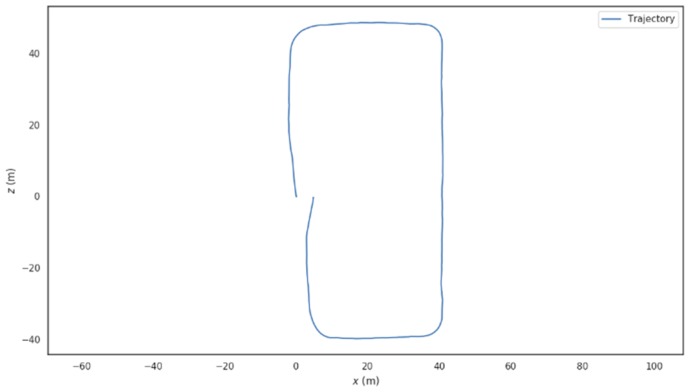
Rectangular trajectory drawn according to the camera motion.

The feature extraction results of the VIO front-end in the substation scene is shown in Figure 13; obviously, the VIO front-end is capable of acquiring abundant point and line features even in cases where the illumination changes frequently (the snow diffuse reflection happens). As in Figure 14, the trajectory drawn according to the camera motion is rectangle distributed, which favorably conforms to the planar geometric appearance of the substation. The fused line features is therefore proven to improve the VIO accuracy both for translation and rotation, and to further improve the VIO robustness under the un-ideal illumination environments.

## 6. Conclusions

An optimized tightly-coupled VIO model which combines an efficient initializing strategy and fused point and line feature matching ideas was employed for navigating and mapping tasks of patrol robots in substations. After exhibiting favorable performances in initializing efficiency, pose estimation and trajectory tracking in a public dataset, this was further experimentally assessed by a campus substation application. It illustrated that, for the feature extraction and matching tasks in the VIO front-end, the fused point and line based method is generally preferred with an L-M optimization strategy; the optimized VIO presents its superiorities even though it is dealing with datasets with different difficulty levels. With respect to the point features and line features, the sparse maps are constructed under the sliding window optimization model, providing the VIO with a necessary location, navigation and obstacle avoidance references. The experimental results showed that a shortened initialization time was derived in practice and that the designed VIO could still accurately fulfill the point and line feature extractions and recover the motion trajectory under un-ideal illumination circumstances. The proposed VIO model therefore fairly meets the SLAM requirements with no external absolute location reference supports.

## Figures and Tables

**Table 1:** The comparative pose measurement results.

		Simple Point Feature-Matching Based Method	Fused Point and Line Feature-Matching Based Method	Direct Method
Non optimization	$T_{F_{1}F_{2}}$	$\left[\begin{array}{cccc} 0.9973 & -0.033 & -0.0647 & -0.0808\\ 0.0343 & 0.9992 & 0.0199 & -0.0858\\ 0.0639 & -0.0221 & 0.9977 & 0.993\\ 0 & 0 & 0 & 1 \end{array}\right]$	$\left[\begin{array}{cccc} 0.9979 & -0.0379 & -0.0514 & -0.1126\\ 0.0397 & 0.9986 & 0.0355 & -0.1137\\ 0.0499 & -0.0374 & 0.998 & 0.2248\\ 0 & 0 & 0 & 1 \end{array}\right]$	×
	RMSE	0.7329	0.2128	-
G-N for 1 round	$T_{F_{1}F_{2}}$	×	$\left[\begin{array}{cccc} 0.998 & -0.0379 & -0.0514 & -0.1125\\ 0.0397 & 0.9986 & 0.03533 & -0.1127\\ 0.0499 & -0.0373 & 0.998 & 0.2248\\ 0 & 0 & 0 & 1 \end{array}\right]$	×
	RMSE	-	0.1020	-
G-N for convergence achieved	$T_{F_{1}F_{2}}$	$\left[\begin{array}{cccc} 0.998 & -0.0373 & -0.0516 & -0.1045\\ 0.04 & 0.9985 & 0.037 & -0.1198\\ 0.05 & -0.039 & 0.998 & 0.2334\\ 0 & 0 & 0 & 1 \end{array}\right]$	$\left[\begin{array}{cccc} 0.9979 & -0.0373 & -0.0516 & -0.1045\\ 0.0392 & 0.9985 & 0.0371 & -0.1198\\ 0.0502 & -0.039 & 0.9979 & 0.2334\\ 0 & 0 & 0 & 1 \end{array}\right]$	$\left[\begin{array}{cccc} 0.9999 & -0.0037 & -0.0005 & 0.0035\\ 0.0034 & 0.9999 & -0.0005 & 0.002\\ 0.0004 & 0.0005 & 1 & -0.0005\\ 0 & 0 & 0 & 1 \end{array}\right]$
	RMSE	0.1010	0.1009	0.2926
L-M for 1 round	$T_{F_{1}F_{2}}$	$\left[\begin{array}{cccc} 0.9973 & -0.0368 & 0.0623 & -0.0325\\ 0.0383 & 0.999 & 0.0223 & -0.0303\\ 0.0614 & -0.0247 & 0.9978 & ,0.234\\ 0 & 0 & 0 & 1 \end{array}\right]$	$\left[\begin{array}{cccc} 0.998 & -0.0373 & -0.0516 & -0.1045\\ 0.03919 & 0.999 & 0.0371 & -0.1198\\ 0.0502 & -0.0391 & 0.9979 & 0.2334\\ 0 & 0 & 0 & 1 \end{array}\right]$	$\left[\begin{array}{cccc} 0.9999 & -0.0037 & -0.0022 & 0.0282\\ 0.0037 & 0.9999 & -0.0001 & 0.003\\ 0.0022 & 0.001 & 0.9999 & -0.044\\ 0 & 0 & 0 & 1 \end{array}\right]$
	RMSE	0.1320	0.1011	0.3376
L-M for convergence achieved	$T_{F_{1}F_{2}}$	$\left[\begin{array}{cccc} 0.9999 & -0.0372 & -0.0516 & -0.1045\\ 0.0392 & 0.9985 & 0.037 & -0.1198\\ 0.0502 & -0.039 & 0.9989 & 0.2334\\ 0 & 0 & 0 & 1 \end{array}\right]$	$\left[\begin{array}{cccc} 0.9978 & -0.0368 & -0.0623 & -0.0325\\ 0.0383 & 0.999 & 0.0224 & -0.0304\\ 0.0614 & -0.0247 & 0.9987 & 0.2341\\ 0 & 0 & 0 & 1 \end{array}\right]$	$\left[\begin{array}{cccc} 0.9999 & -0.0034 & -0.0023 & 0.0286\\ 0.0038 & 0.9999 & -0.0008 & 0.0016\\ 0.0023 & 0.0008 & 0.9999 & -0.0448\\ 0 & 0 & 0 & 1 \end{array}\right]$
	RMSE	0.0601	0.0486	0.2917

**Table 2:** The comparative time consumption results (s).

	Feature Extraction	Descriptor Calculation	Feature Matching	Pose Estimation	Total
ORB SURF SIFT	0.0101 0.0435 0.9228	0.0087 0.0095 0.0125	0.0118 0.0274 0.0285	0.0009 0.0014 0.0012	0.0315 0.0818 0.9650

**Table 3:** The comparative absolute positioning errors in the European Robotics Challenge (EUROC) datasets.

	Ref. [42]	Ref. [25]	Ref. [18]	Ref. [35]	Ref. [39]	VIO Designed
V1_01_easy	0.1167	0.0958	0.0716	0.0544	0.0591	0.0524
V1_02_medium	0.1392	0.0964	0.0912	0.0849	0.0766	0.0724
V1_03_diffcult	0.1934	×	0.1742	0.1597	0.1302	0.1102
V2_01_easy	0.1267	0.0858	0.1017	0.0712	0.0502	0.0413
V2_02_medium	0.2049	0.1525	0.1876	0.1638	0.0945	0.0815
V2_03_diffcult	×	0.2588	0.2719	0.2347	0.2609	0.2176
MH_01_easy	0.2557	0.1537	0.1647	0.1221	0.0731	0.0513
MH_02_easy	0.1861	0.1595	0.1573	0.1287	0.2327	0.0407
MH_03_medium	0.2176	0.1719	0.2077	0.1365	0.1122	0.1065
MH_04_diffcult	0.3037	0.3165	0.3921	0.1894	0.1394	0.1377
MH_05_diffcult	0.3509	×	×	0.2173	0.2569	0.1546

**Table 4:** Calibration parameters of camera and IMU.

Camera Intrinsic	Focal length: $f_{x}=363.034$ pixel, $f_{y}=364.019$ pixel Principal point of photograph: [366.871, 243.308] Radial distortion: [−3.08252, 8.42513, −1.50093, 2.01707]
Camera/IMU Extrinsic	$T_{CB}=\left[\begin{array}{cccc} -0.00647 & -0.99995 & -0.00764 & 0.00534\\ 0.99998 & -0.00647 & -0.00009 & -0.04303\\ 0.00005 & -0.00764 & 0.99997 & 0.02303\\ 0 & 0 & 0 & 1 \end{array}\right]$
Image parameters	Image resolution: 752 × 480 pixel
